# Supplementary material for: Evidence for saddle point-driven charge density wave on the surface of heavily hole-doped iron arsenide superconductors
Source: Nat Commun. 2025 Jan 2;16:253. doi: 10.1038/s41467-024-55368-7 (PMC11695596; doi:10.1038/s41467-024-55368-7)
Supplement: Supplementary file 1 — Supplementary Information [file 41467_2024_55368_MOESM1_ESM.pdf]

Supplementary Information for 'Evidence for saddle point-driven charge density wave on the surface of heavily hole-doped iron arsenide superconductors'

Quanxin Hu*,[1] Yu Zheng*,[1] Hanxiang Xu,[2] Junze Deng,[2,3] Chenhao Liang,[2,3] Fazhi Yang,[1] Zhijun Wang,[2,3] Vadim Grinenko,[1] Baiqing Lv,[1,4,†] Hong Ding,[1,4,5,‡] and Chi Ming Yim[1,§]

[1]Tsung-Dao Lee Institute and School of Physics and Astronomy, Shanghai Jiao Tong University, Shanghai 201210, China

[2]Beijing National Laboratory for Condensed Matter Physics, Institute of Physics, Chinese Academy of Sciences, Beijing 100190, China

[3]School of Physical Sciences, University of Chinese Academy of Sciences, Beijing 100049, China

[4]Hefei National Laboratory, Hefei 230088, China

[5]New Cornerstone Science Laboratory, Shanghai 201210, China

(Dated: December 6, 2024)

* These authors contributed equally.

[†]Electronic address: baiqing@sjtu.edu.cn

[‡]Electronic address: dingh@sjtu.edu.cn

[§]Electronic address: c.m.yim@sjtu.edu.cn

This Supplementary Information contains Supplementary Notes 1-2 and Supplementary Figures S1-S12.

Supplementary Note 1. Identifying the As-terminated surface of $Ba_{1-x}K_xFe_2As_2$

Due to the fact that the image contrast in the constant current topographic images originates from the convolution between the integrated local density of states and the geometrical corrugations, simply assigning the surface terminations of $Ba_{1-x}K_xFe_2As_2$ by their apparent heights would not be appropriate. However, tip-induced aggregation of Ba/K surface adatoms provides a convenient way for identifying surfaces of different terminations. In the STM topographic image obtained from the surface of a freshly cleaved sample of KFe_2As_2 in Fig. S2, the upper terraces in the top-left and bottom-right corners exhibit a $\sqrt{2} \times \sqrt{2}$ unit cell (not shown). According to earlier STM studies, they can be assigned to the $\sqrt{2} \times \sqrt{2}$ reconstruction of the K-terminated surface. On the other hand, the lower terrace in the central part of the image comprises a large number of randomly scattered protrusions. These protrusions are highly mobile and can be displaced to the edge(s) of the imaged area using the STM tip. In addition, once they aggregate, they form a new surface with a measured height consistent with the $\sqrt{2} \times \sqrt{2}$ K-terminated surface. On this basis, we assign the protrusions as K adatoms left on the surface as a result of sample cleaving, and the lower terrace where the K adatoms reside as the As-terminated terrace. An adatom-free As-terminated surface region can be formed by tip-induced displacement of all K adatoms from the imaged region, see Figs. S1 and S2b.

Supplementary Note 2. Examination of the As-terminated surfaces of samples with other K-doping levels

To determine the origin of the charge density wave (CDW), we also examined the As-terminated surfaces of KFe_2As_2 and $Ba_{0.55}K_{0.45}Fe_2As_2$. As shown in Fig. S6, CDW persists on the As-terminated surface of KFe_2As_2 , but not on that of $Ba_{0.55}K_{0.45}Fe_2As_2$.

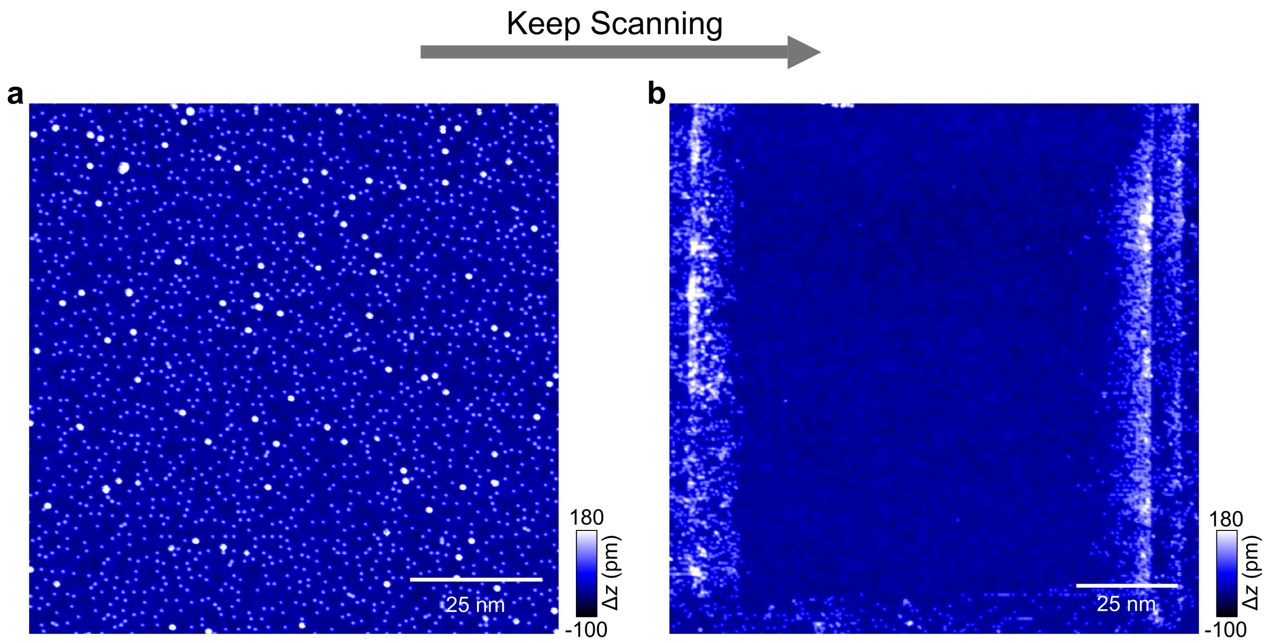

Fig. S1: **Preparation of an adatom-free As-terminated surface of $\mathbf{Ba_{1-x}K_xFe_2As_2}$** $(x \approx 0.77)$ **through tip-induced displacement of Ba/K surface adatoms.** **a**, Topographic image of the As-terminated surface of a freshly cleaved $Ba_{1-x}K_xFe_2As_2$ $(x \approx 0.77)$ sample $[(V, I) = (80\,\mathrm{mV}, 100\,\mathrm{pA})$, image size: $(100\,\mathrm{nm})^2]$. The surface is populated with randomly scattered Ba/K adatoms left on the surface as a result of sample cleaving. **b**, As (**a**), recorded after all Ba/K adatoms were displaced to the edges of the imaged region by the STM tip [image size: $(130\,\mathrm{nm})^2$].

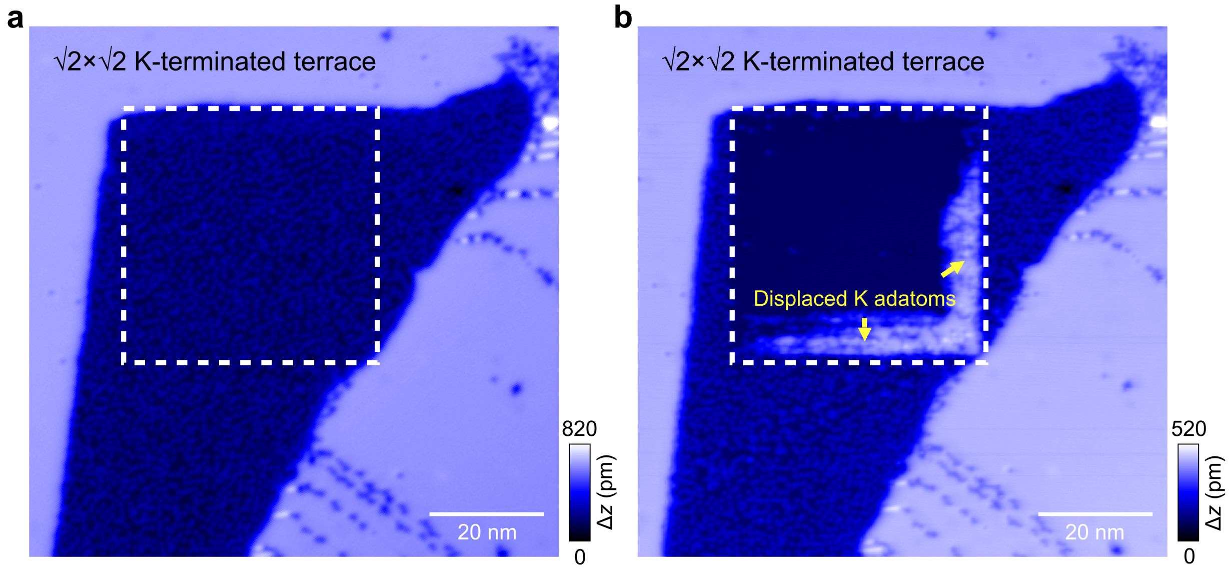

Fig. S2: **Preparation of an adatom-free As-terminated surface of KFe_2As_2 through tip-induced displacement of K surface adatoms.** **a**, STM topographic image taken from the surface of a freshly cleaved KFe_2As_2 sample $[(V, I) = (100\,\mathrm{mV}, 300\,\mathrm{pA})$, image size: $(92.3\,\mathrm{nm})^2]$. The imaged region comprises $(\sqrt{2}\times\sqrt{2})$-reconstructed K-terminated upper terraces in the top-left and bottom-right corners, and a As-terminated lower terrace at the centre. The As-terminated terrace is also populated with randomly scattered K adatoms left as a result of sample cleaving. **b**, As (**a**), recorded after the K adatoms were displaced by the STM tip to the edges of a smaller imaged region (marked by a dashed square) $[(V, I) = (80\,\mathrm{mV}, 100\,\mathrm{pA})$, image size: $(92.3\,\mathrm{nm})^2]$.

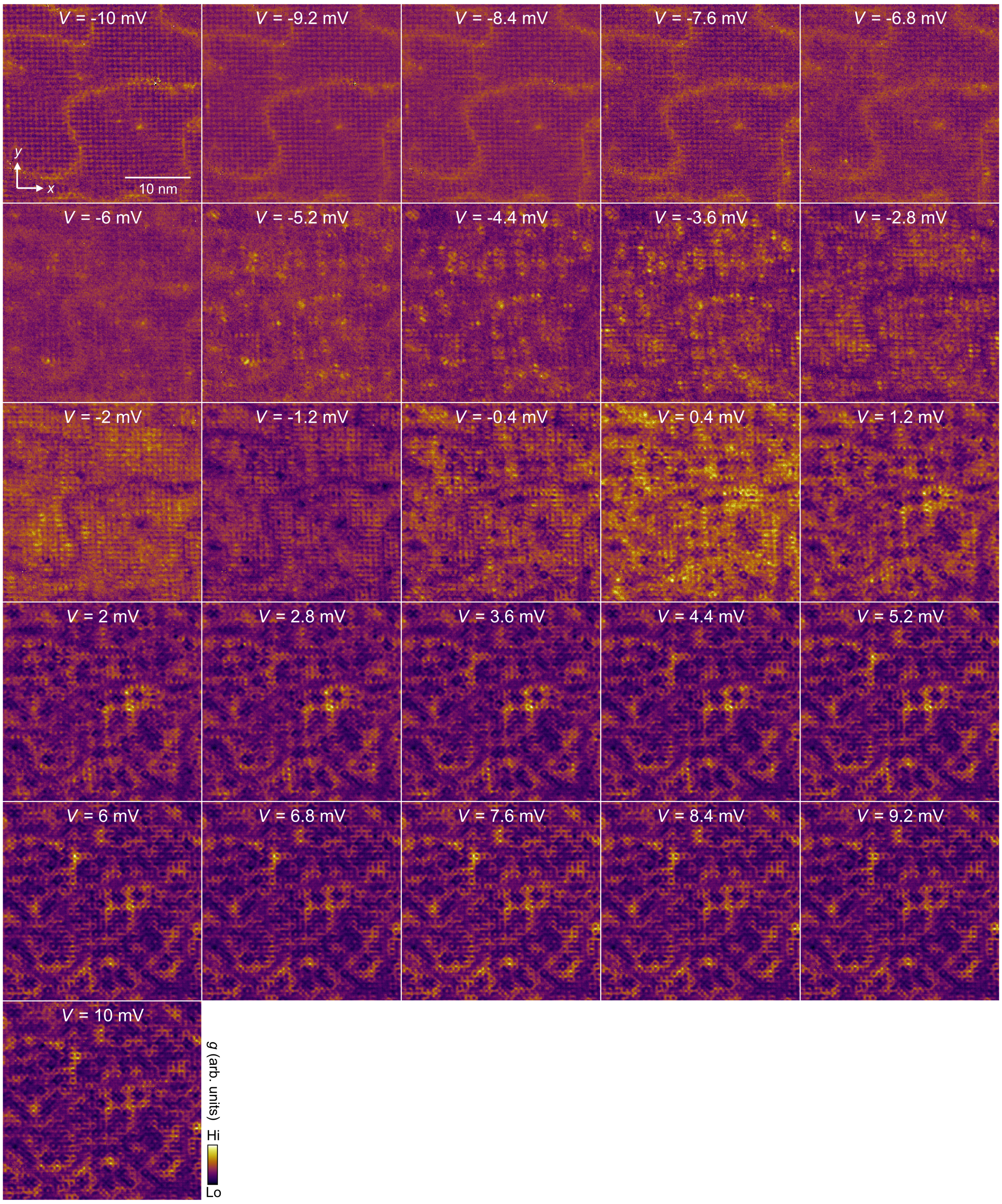

Fig. S3: $\mathrm{d}I/\mathrm{d}V(\mathbf{r}, V)$ **map recorded from the As-terminated surface of** $\mathbf{Ba_{1-x}K_xFe_2As_2}$ $(x \approx 0.77)$**.** Spectroscopy set-points: $(V_s, I_s) = (-10\,\mathrm{mV}, 100\,\mathrm{pA})$. Amplitude of bias modulation used $V_{\mathrm{mod}} = 0.25\,\mathrm{mV}$. Image size: $(30\,\mathrm{nm})^2$.

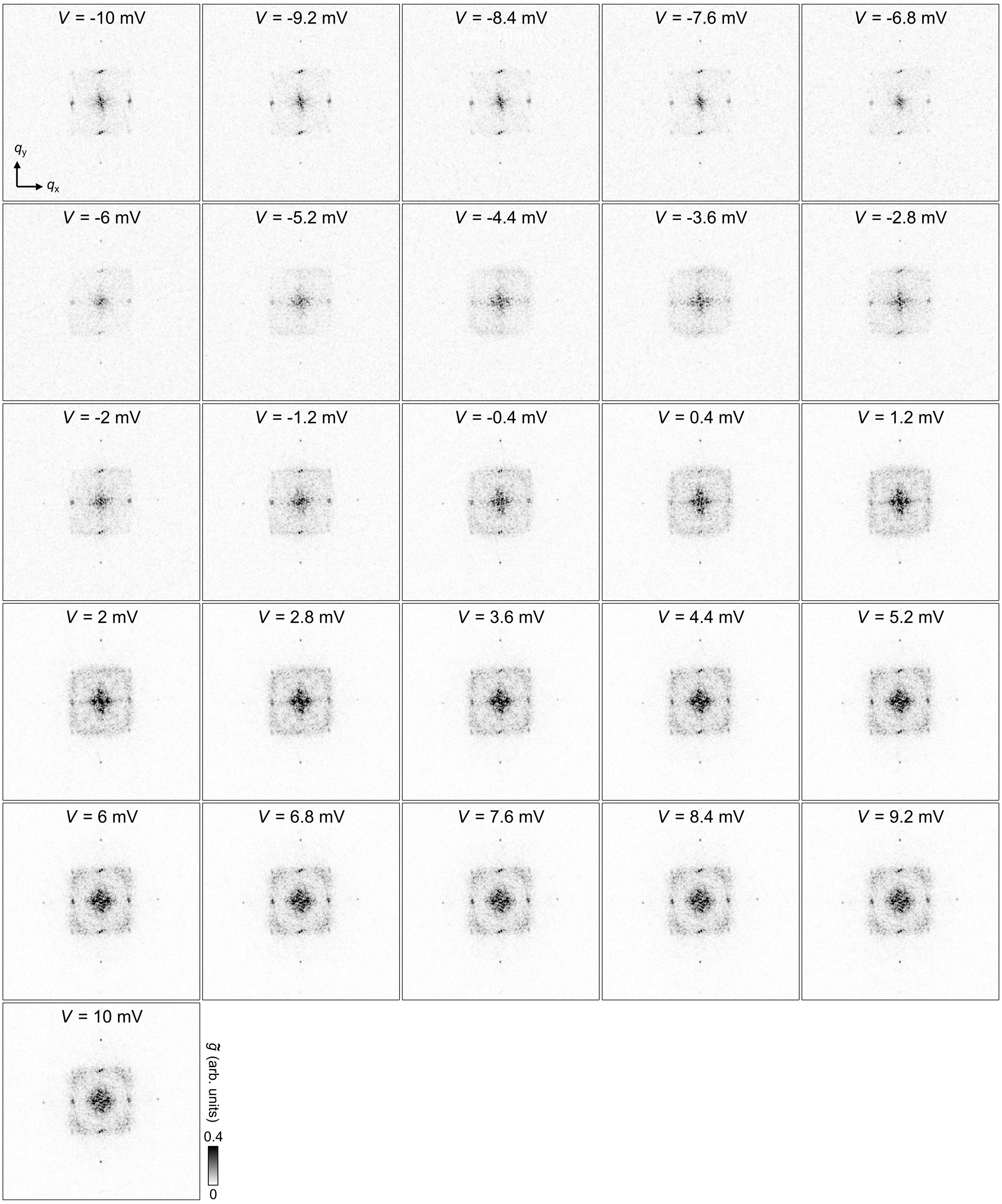

Fig. S4: $\tilde{g}(\mathbf{q}, V)$ **map of the As-terminated surface of** $\mathbf{Ba_{1-x}K_xFe_2As_2}$ **(**$x \approx 0.77$**).** Generated by Fourier transformation (without distortion correction or symmetrization) on the real space $\mathrm{d}I/\mathrm{d}V(\mathbf{r}, V)$ map in Fig. S3.

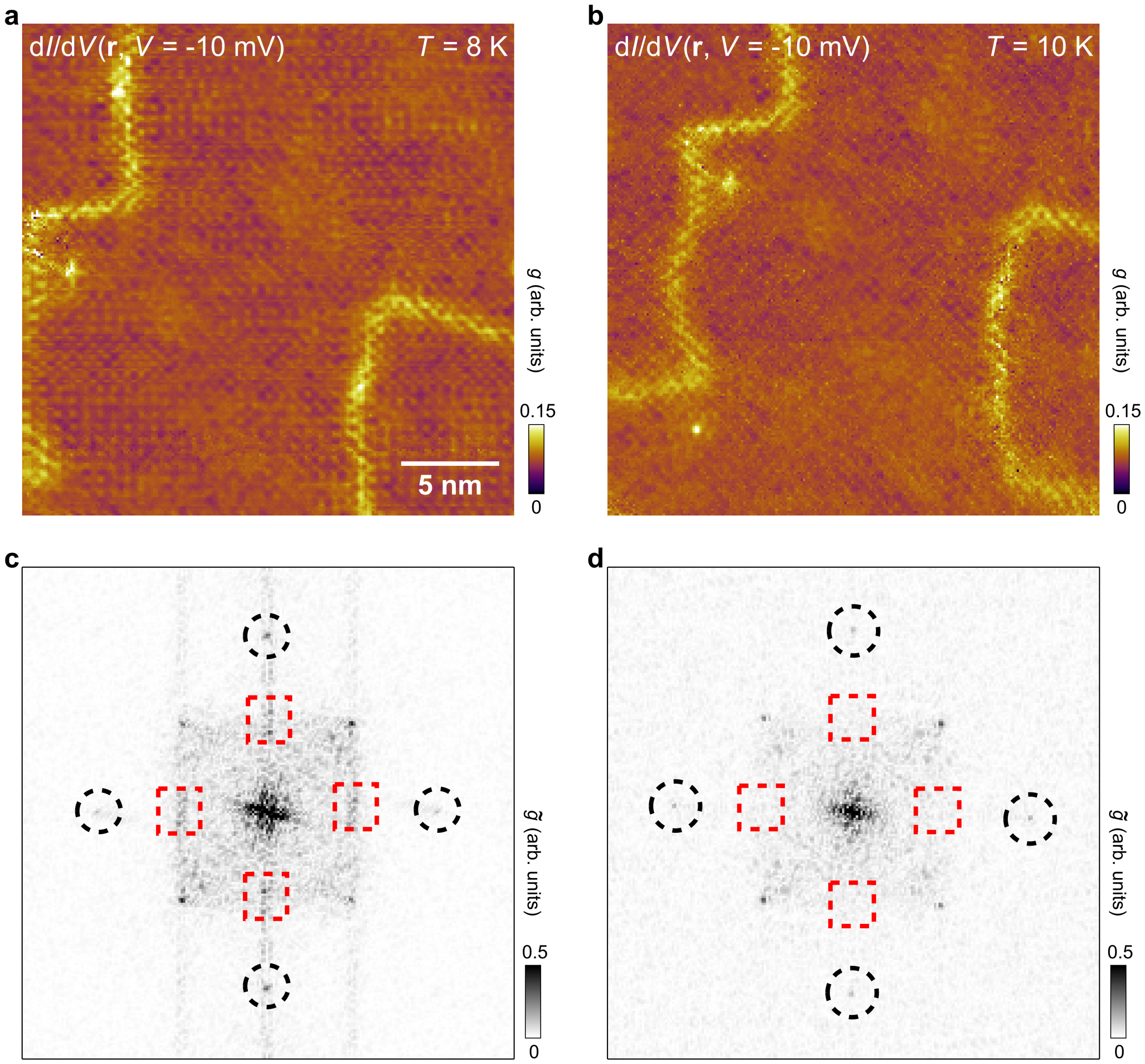

Fig. S5: **Comparison of differential conductance maps recorded using two different map acquisition modes. a-b**, Constant-height $\mathrm{d}I/\mathrm{d}V(\mathbf{r}, V)$ map slices at $V = -10\,\mathrm{mV}$ obtained from the same surface location as Fig. 3 at temperatures of (**a**) 8 and (**b**) $10\,\mathrm{K}$ respectively $[(V_s, I_s) = (-10\,\mathrm{mV}, 300\,\mathrm{pA})$, image size: $(25\,\mathrm{nm})^2]$. **c-d**, Fourier Transformations of (**a**) and (**b**). Thermal drift became a problem in map data acquisition at increased temperatures. To minimize its effect, the $\mathrm{d}I/\mathrm{d}V_{\mathrm{cc}}(\mathbf{r}, V)$ map data in Fig. 3 were recorded in the constant current spectroscopic imaging mode, during which the current feedback loop was kept closed. Through comparison of the map slices here with those in Fig. 3, we confirm that $\mathrm{d}I/\mathrm{d}V_{\mathrm{cc}}(\mathbf{r}, V)$ mapping does not lead to any undesired artifact.

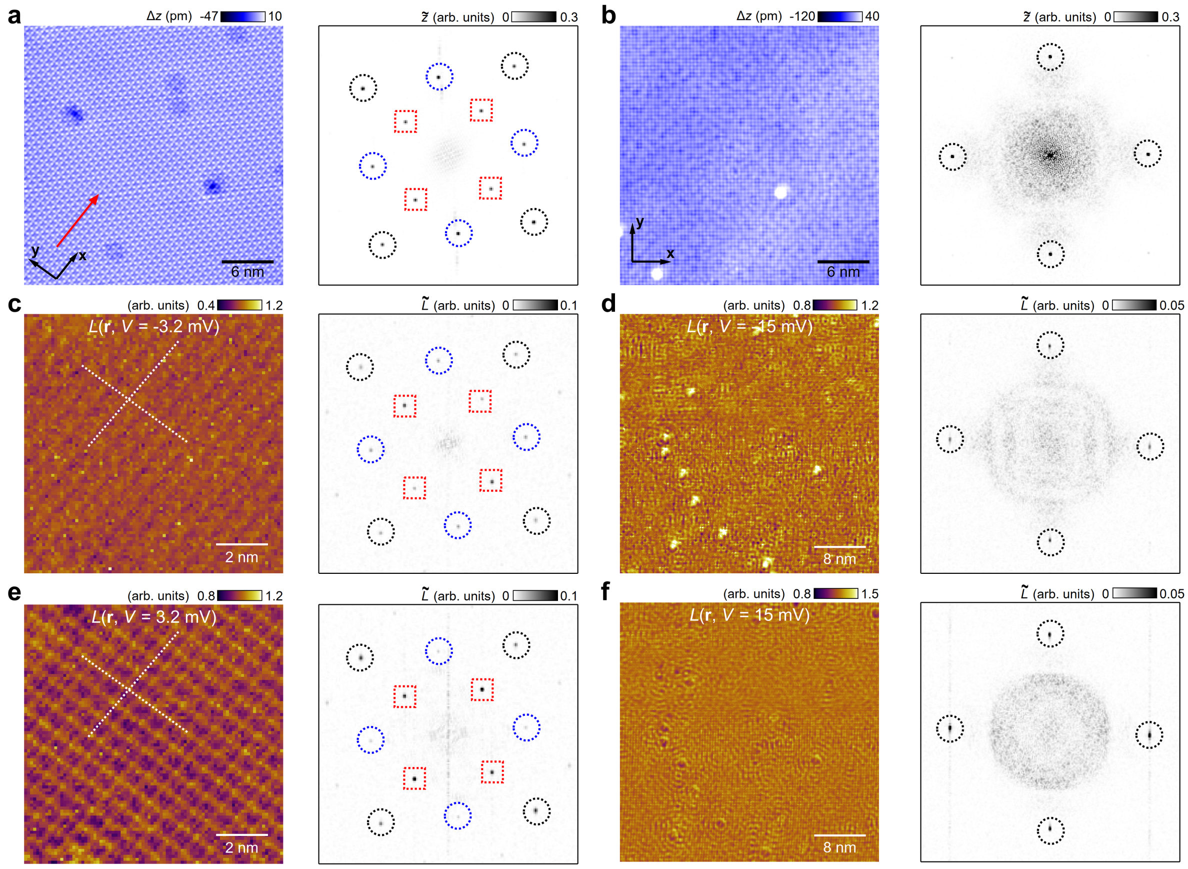

Fig. S6: **Topographic images and spectroscopic map data of the As-terminated surfaces of $\mathbf{KFe_2As_2}$ and $\mathbf{Ba_{1-x}K_xFe_2As_2}$** ($x \approx 0.45$)**. a**, **b**, Topographic images and their Fourier transformations recorded from the As-terminated surfaces of (**a**) KFe_2As_2 [$(V, I) = (20\,\mathrm{mV}, 200\,\mathrm{pA})$, image size: $(30\,\mathrm{nm})^2$] and (**b**) $Ba_{1-x}K_xFe_2As_2$ ($x \approx 0.45$) [$(V, I) = (-10\,\mathrm{mV}, 100\,\mathrm{pA})$, image size: $(30\,\mathrm{nm})^2$]. **c**, **e**, Normalised differential conductance $L(\mathbf{r}, V)$ map slices at bias voltages of (**c**) -3.2 and (**e**) $+3.2\,\mathrm{mV}$ obtained from a $(10\,\mathrm{nm})^2$ area on the As-terminated surface of KFe_2As_2 [$(V_s, I_s) = (20\,\mathrm{mV}, 500\,\mathrm{pA})$, $V_{\mathrm{mod}} = 1.25\,\mathrm{mV}$] and their Fourier transformations. **d**, **f**, $L(\mathbf{r}, V)$ map slices at bias voltages of (**d**) -15 and (**f**) $+15\,\mathrm{mV}$ obtained from a $(40\,\mathrm{nm})^2$ area on that of $Ba_{1-x}K_xFe_2As_2$ ($x \approx 0.45$) [$(V_s, I_s) = (-30\,\mathrm{mV}, 500\,\mathrm{pA})$, $V_{\mathrm{mod}} = 1.75\,\mathrm{mV}$] and their Fourier transformations.

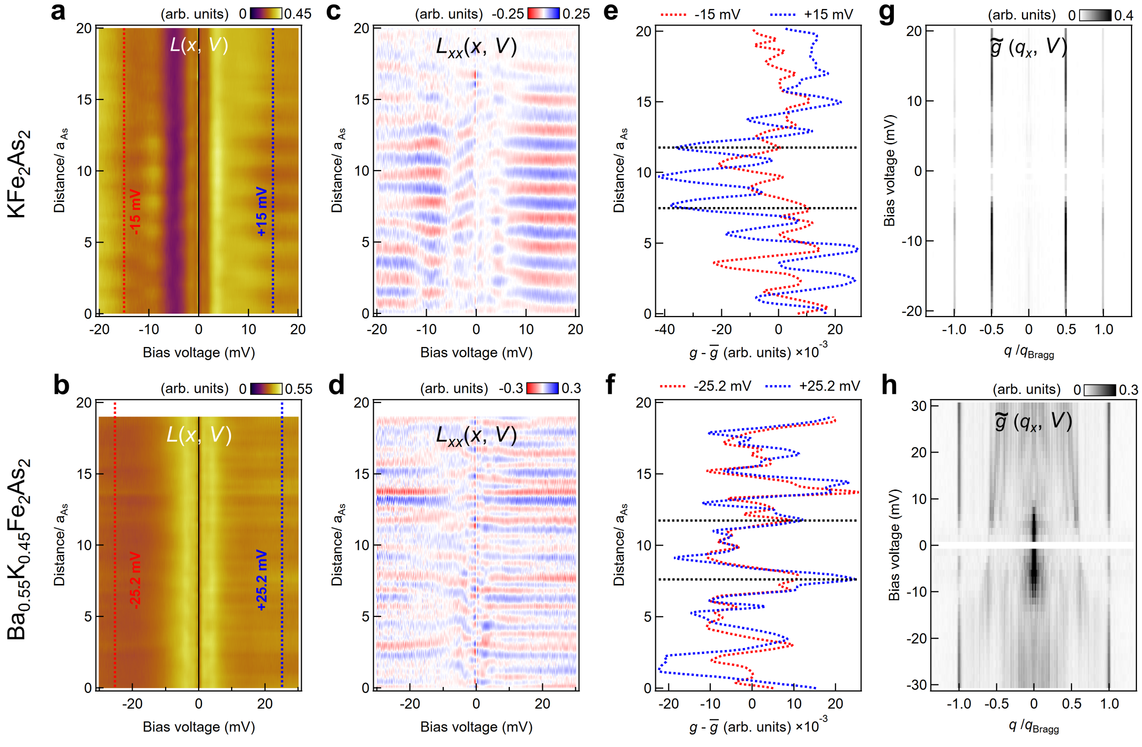

Fig. S7: Spectroscopic characteristics of the As-terminated surfaces of $\mathbf{KFe_2As_2}$ and $\mathbf{Ba_{1-x}K_xFe_2As_2}$ ($x \approx 0.45$). a, **b**, Normalised differential conductance spectra $L(x, V)$ taken along the As-As direction on the As-terminated surfaces of (**a**) KFe_2As_2 $[(V_s, I_s) = (20\,\mathrm{mV}, 500\,\mathrm{pA}),\ V_{\mathrm{mod}} = 0.25\,\mathrm{mV}]$ and (**b**) $Ba_{1-x}K_xFe_2As_2$ ($x \approx 0.45$) $[(V_s, I_s) = (-30\,\mathrm{mV}, 500\,\mathrm{pA}),\ V_{\mathrm{mod}} = 0.5\,\mathrm{mV}]$. **c**, **d**, Second derivatives of (**a**) and (**b**) with respect to position. **e**, $L(x)$ plots at bias voltages of -15 (red) and $+15\,\mathrm{mV}$ (blue) extracted from (**a**), with each plot subtracted by its respective spatial average. **f**, $L(x)$ plots at bias voltages of -25.2 (red) and $+25.2\,\mathrm{mV}$ (blue) extracted from (**b**), with each plot subtracted by its respective spatial average. **g**, $\tilde{g}(q_x, V)$ along $\Gamma - \mathrm{M}$ plot on the As-terminated surface of KFe_2As_2 showing non-dispersive behavior of the charge order peaks. **h**, $\tilde{g}(q_x, V)$ along $\Gamma - \mathrm{M}$ plot on that of $Ba_{1-x}K_xFe_2As_2$ ($x \approx 0.45$) showing dispersive quasi-particle scattering behavior.

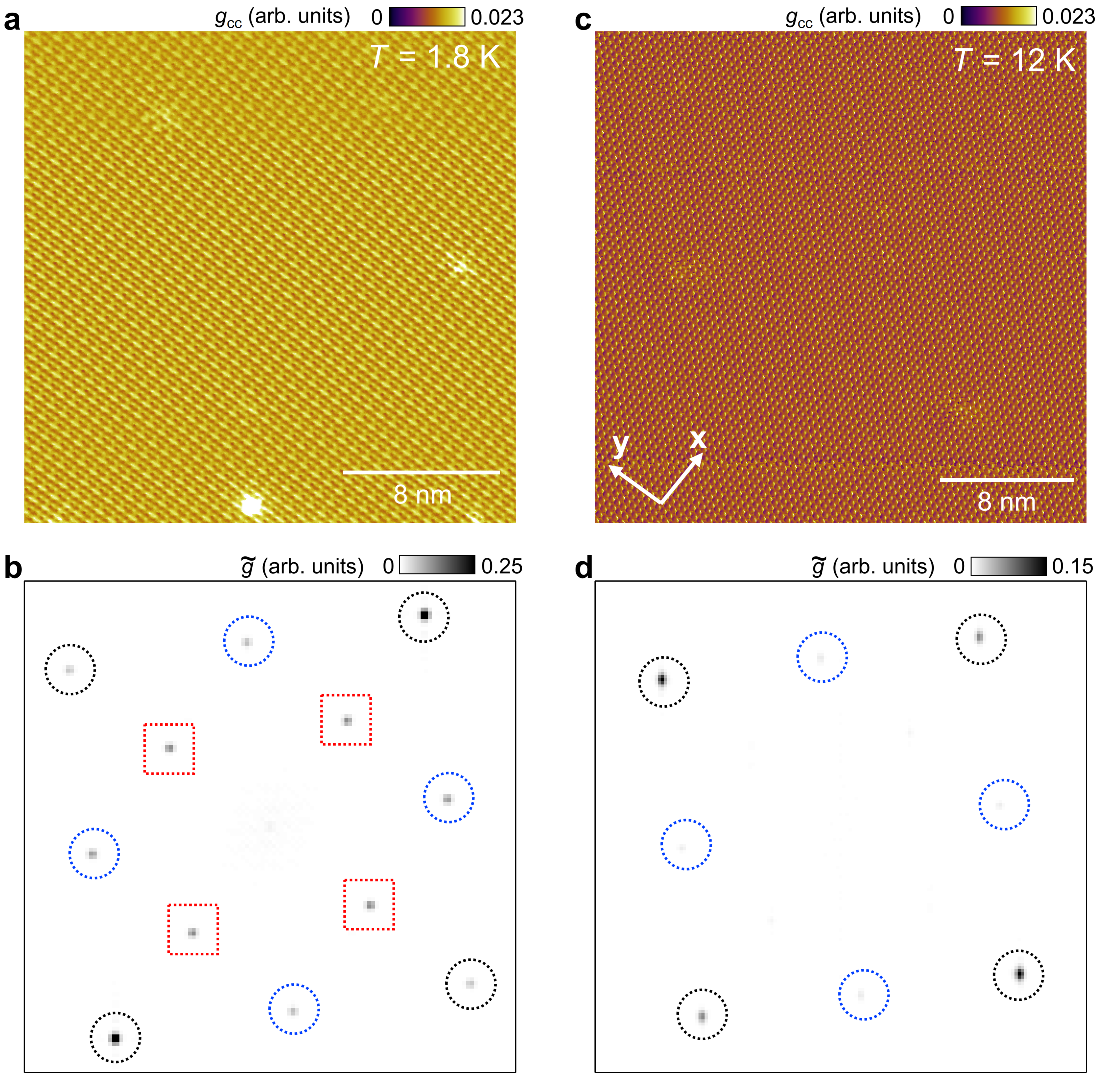

Fig. S8: CDW order on the As-terminated surface of KFe_2As_2 . a, c, $\mathrm{d}I/\mathrm{d}V_{cc}(\mathbf{r}, V)$ map slides at $V = -10\,\mathrm{mV}$ recorded from the As-terminated surface of KFe_2As_2 at temperatures of (**a**) $1.8\,\mathrm{K}$ and (**c**) $12\,\mathrm{K}$ $[(V_s, I_s) = (-10\,\mathrm{mV}, 200\,\mathrm{pA}), V_{\mathrm{mod}} = 0.75\,\mathrm{mV}]$. **b, d**, Fourier transformations of (**a**) and (**c**). The data shows that the (2×2) CDW order observed at $1.8\,\mathrm{K}$ vanishes at $12\,\mathrm{K}$.

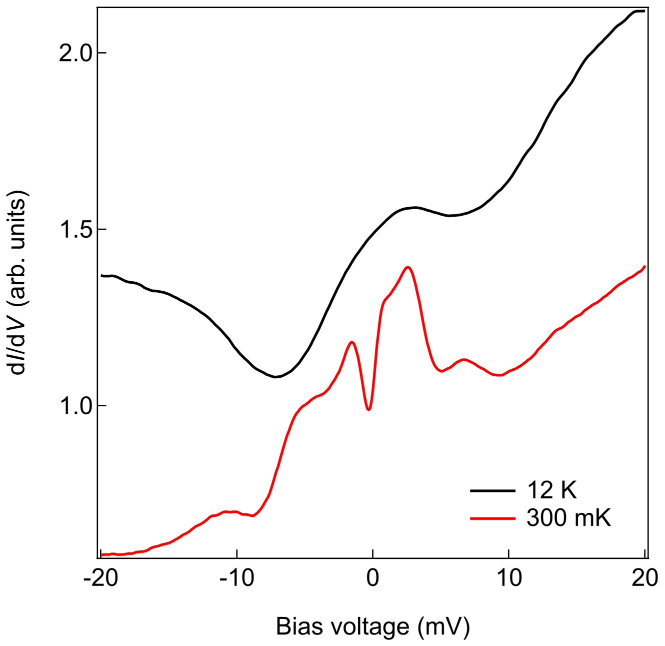

Fig. S9: **Point tunnelling spectra taken from the As-terminated surface of KFe_2As_2 at two different temperatures:** (red) $0.3\,\mathrm{K}$ [$(V_s, I_s) = (20\,\mathrm{mV}, 500\,\mathrm{pA})$, $V_{\mathrm{mod}} = 0.25\,\mathrm{mV}$] and (black) $12\,\mathrm{K}$ [$(V_s, I_s) = (-20\,\mathrm{mV}, 400\,\mathrm{pA})$, $V_{\mathrm{mod}} = 0.25\,\mathrm{mV}$]. The spectra are vertically offset for clarity. The $0.3\,\mathrm{K}$ spectrum exhibits a CDW gap extending between -1.5 and $+1\,\mathrm{meV}$ in energy, which disappears at $12\,\mathrm{K}$.

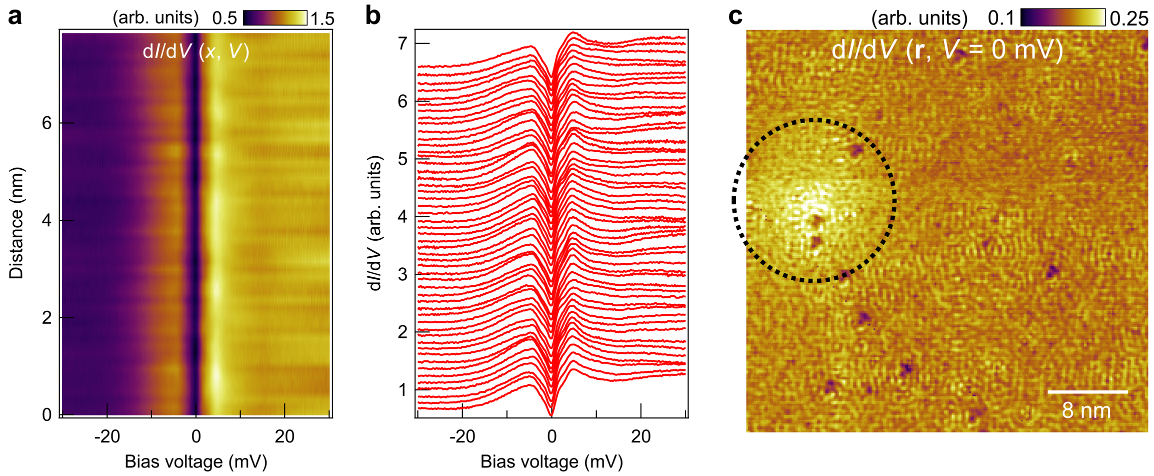

Fig. S10: **Superconductivity on the As-terminated surface of $\mathbf{Ba_{1-x}K_xFe_2As_2}$** $(x \approx 0.45)$. **a**, $\mathrm{d}I/\mathrm{d}V(x, V)$ plot recorded from the As-terminated surface of $Ba_{1-x}K_xFe_2As_2$ $(x \approx 0.45)$ $[(V_s, I_s) = (-30\,\mathrm{mV}, 500\,\mathrm{pA}), V_{\mathrm{mod}} = 0.5\,\mathrm{mV}]$. **b**, Corresponding waterfall plot of (**a**). **c**, Zero-bias conductance map measured in a $(40\,\mathrm{nm})^2$ area at $300\,\mathrm{mK}$ with an applied magnetic field of $0.5\,\mathrm{T}$ $[(V_s, I_s) = (-30\,\mathrm{mV}, 500\,\mathrm{pA}), V_{\mathrm{mod}} = 1.75\,\mathrm{mV}]$, showing the presence of a superconducting vortex in the imaged region (marked by the black dashed circle).

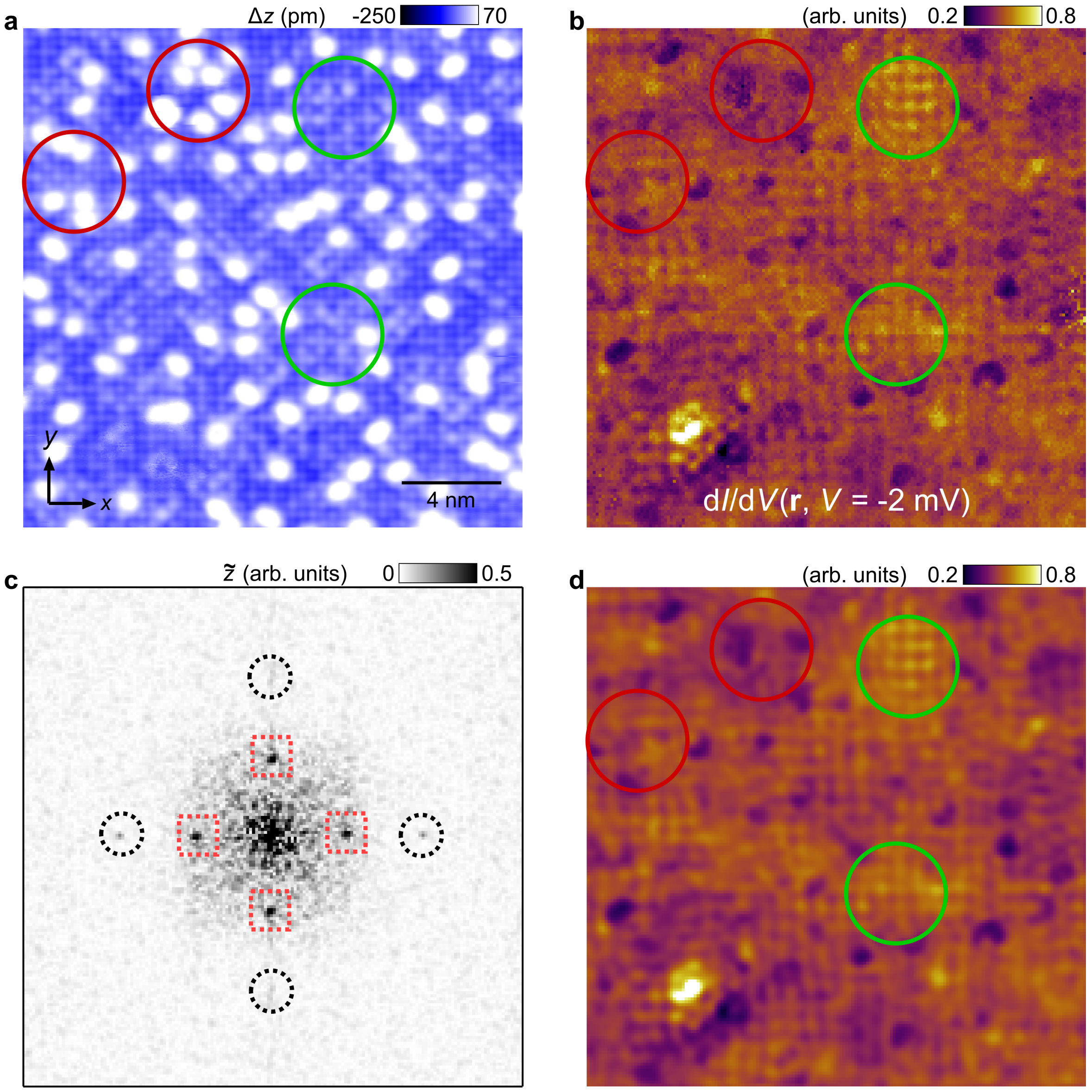

Fig. S11: Doping effect on the CDW order. **a**, Atomically-resolved topographic image of As-terminated surface of $Ba_{1-x}K_xFe_2As_2$ $(x \approx 0.77)$ obtained before Ba/K surface adatoms were removed $[V = -10\,\mathrm{mV},\ I = 100\,\mathrm{pA}$, image size: $(20\,\mathrm{nm})^2]$. Ba/K adatoms act as electron donors to the surface. Red (green) circles mark the adatom-dense (-free) regions. **b**, $\mathrm{d}I/\mathrm{d}V(\mathbf{r}, V)$ map slice at $V = -2\,\mathrm{mV}$ obtained at the same surface location as (**a**), showing presence of the CDW order in the adatom-free regions only. **c**, Fourier transformation of (**b**). Black circles highlight the Bragg peaks, red squares the (2×2) CDW order peaks. **d**, Box-filtered map of (**b**) generated from the (2×2) CDW order peaks, where the checkerboard-like patterns become more apparent.

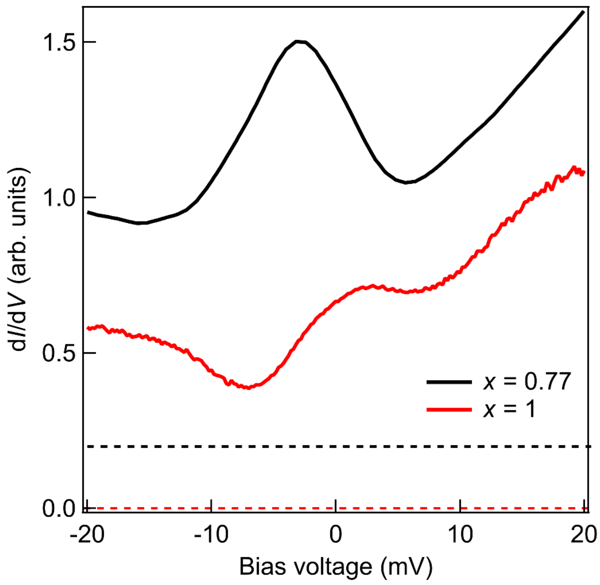

Fig. S12: **Normal-state differential conductance spectra of the As-terminated surfaces of $Ba_{1-x}K_xFe_2As_2$ samples with two different levels of K-doping:** (black) $x = 0.77$ and (red) $x = 1$. Spectroscopy setpoints: $[(V_s, I_s) = (-10\,\mathrm{mV}, 200\,\mathrm{pA}), V_{\mathrm{mod}} = 0.25\,\mathrm{mV}]$ for the black curve, and $[(V_s, I_s) = (-20\,\mathrm{mV}, 400\,\mathrm{pA}), V_{\mathrm{mod}} = 0.25\,\mathrm{mV}]$ for the red curve. Both spectra were recorded at a temperature of $12\,\mathrm{K}$. Spectra are vertically offset for clarity. Dashed lines of black and red color represent the respective zero conductance.
